# The Eat Smart Study: A randomised controlled trial of a reduced carbohydrate versus a low fat diet for weight loss in obese adolescents

**DOI:** 10.1186/1471-2458-10-464

**Published:** 2010-08-09

**Authors:** Helen Truby, Kimberley A Baxter, Paula Barrett, Robert S Ware, John C Cardinal, Peter SW Davies, Lynne A Daniels, Jennifer A Batch

**Affiliations:** 1 Department of Nutrition and Dietetics, Monash University, Victoria, 3168, Australia; 2 Children's Nutrition Research Centre, Royal Children's Hospital, Herston, Queensland, 4029, Australia; 3 Pathways Health and Research Centre, 88 Boundary Street, West End, Queensland, 4102, Australia; 4 School of Education, University of Queensland, Queensland, 4029, Australia; 5 School of Population Health, University of Queensland, Queensland, 4029, Australia; 6 Queensland Children's Medical Research Institute, Herston, Queensland, 4029, Australia; 7 Chemical Pathology, Pathology Queensland, Herston, Queensland 4029, Australia; 8 Institute of Health and Biomedical Innovation Health, School of Public Health, Queensland University of Technology, Kelvin Grove, Queensland, 4001, Australia; 9 Department of Endocrinology and Diabetes, Royal Children's Hospital, Herston, Queensland, 4029, Australia

## Abstract

**Background:**

Despite the recognition of obesity in young people as a key health issue, there is limited evidence to inform health professionals regarding the most appropriate treatment options. The Eat Smart study aims to contribute to the knowledge base of effective dietary strategies for the clinical management of the obese adolescent and examine the cardiometablic effects of a reduced carbohydrate diet versus a low fat diet.

**Methods and design:**

Eat Smart is a randomised controlled trial and aims to recruit 100 adolescents over a 2 1/2 year period. Families will be invited to participate following referral by their health professional who has recommended weight management. Participants will be overweight as defined by a body mass index (BMI) greater than the 90^th ^percentile, using CDC 2000 growth charts. An accredited 6-week psychological life skills program 'FRIENDS for Life', which is designed to provide behaviour change and coping skills will be undertaken prior to volunteers being randomised to group. The intervention arms include a structured reduced carbohydrate or a structured low fat dietary program based on an individualised energy prescription. The intervention will involve a series of dietetic appointments over 24 weeks. The control group will commence the dietary program of their choice after a 12 week period. Outcome measures will be assessed at baseline, week 12 and week 24. The primary outcome measure will be change in BMI z-score. A range of secondary outcome measures including body composition, lipid fractions, inflammatory markers, social and psychological measures will be measured.

**Discussion:**

The chronic and difficult nature of treating the obese adolescent is increasingly recognised by clinicians and has highlighted the need for research aimed at providing effective intervention strategies, particularly for use in the tertiary setting. A structured reduced carbohydrate approach may provide a dietary pattern that some families will find more sustainable and effective than the conventional low fat dietary approach currently advocated. This study aims to investigate the acceptability and effectiveness of a structured reduced dietary carbohydrate intervention and will compare the outcomes of this approach with a structured low fat eating plan.

**Trial Registration:**

The protocol for this study is registered with the International Clinical Trials Registry (ISRCTN49438757).

## Background

Obesity is being increasingly recognised as a major chronic health problem for children and adolescents [1,2]. A review of worldwide obesity trends in children in 2006, found that there has been a documented increase in obesity in all countries for which data exists [3]. Rates in Australia have also increased over time and the most current estimate from the 2007 Australian Children's Nutrition and Physical Activity Survey, places 23% of Australian young people (aged 2-16 years) in the overweight or obese weight category [4]. The Healthy Kids Queensland Survey in 2006, noted an increase in the proportion of children who are severely obese, demonstrated by BMI and waist circumferences both increasing at the upper end of the distribution [5].

Research has found that being overweight as a child is a strong predictor of being overweight or obese as an adult [6]. Obesity in childhood therefore represents a critical window of opportunity for intervention; not only to address an existing health problem but as a secondary prevention strategy for the array of medical and social consequences associated with obesity over the lifespan. Obesity is a major public health problem with enormous economic consequences. In Australia, for example, estimates of the net cost of obesity in 2008 (excluding overweight) which takes into account individual burden of disease, was estimated to be in excess of $58 billion [7]. Higher BMI in childhood is associated with an increased risk of Coronary Heart Disease (CHD) in adulthood and the longer the child remains overweight the higher the associated risk [8]. Baker et al (2007) reported that for every 1 unit increase in BMI z-score the hazard ratio for boys aged 7 years of a fatal CHD event in adulthood was 1.10 and at age 13 years was 1.24; for girls the hazard ratio was 1.07 at age 7 and 1.23 at age 13 years [8]. These facts support the need to treat the obese child however research into dietary strategies to manage obesity has been limited to date in both quantity and quality [9].

Protein is the most satiating of the macronutrients and there is evidence that increasing protein confers a thermogenic benefit and spares the loss of muscle when an adult is in negative energy balance (i.e. during a weight loss attempt) [10]. The Atkins diet [11] recommends a radical reduction in carbohydrate with no restriction of protein or fat. The Atkins diet was hugely popular but sparked immediate controversy because its macronutrient distribution contravened the accepted approach of a low fat diet and it was thought that a very high fat diet would have adverse effects on cardiovascular health. Emerging evidence in adults shows that reduced carbohydrate diets can result in loss of body fat, improved insulin sensitivity and markers of inflammation compared to conventional approaches [12]. One of the most well known reduced carbohydrate diets in Australia is the CSIRO Total Well Being Diet [13]. Several adult studies have shown its effectiveness in achieving weight loss, reducing body fat and improving biochemical markers of cardiometabolic risk such as reduction in triglycerides, insulin and glucose which are independent of weight loss [14-16].

In 2007 we ran a feasibility study to test the acceptability and effectiveness of a reduced carbohydrate diet (35% carbohydrate, 35% fat) versus a low fat approach (25% fat, 55% carbohydrate). We tested three dietary approaches in this proof of concept phase: an unstructured low fat diet (current 'standard care'), a structured low fat diet using a plate portion system and a semi-structured reduced carbohydrate diet. When given the choice, 10% chose the standard unstructured low fat, 42% the structured low fat, and 48% opted to modify their carbohydrate intake [17]. This demonstrated that adolescents had a preference for structured advice and were willing to modify carbohydrate intake. It highlighted that the current 'standard care' model of generalised lifestyle advice which relied on motivation and self monitoring was not what these young people were seeking. Research in adults has also showed a preference and higher level of efficacy in prescriptive dietary advice as opposed to generalised lifestyle advice [18]. Prior to commencement of the dietary plan of their choice, measurement of food intake showed a typical dietary pattern in adolescents of 37% of energy from fat, 17% from protein and 45% from carbohydrate. Both our dietary plans resulted in reduction in total fat intake (12% in the low fat and 2% in the reduced carbohydrate group). In light of these clear preferences for structured advice we will take forward into the full randomised control trial (RCT) only the structured dietary approaches in order to examine whether change in macronutrient distribution has an effect on cardiovascular risk. The dietary composition will be informed by our findings in our feasibility study which provides proof of concept for these approaches [19].

In our feasibility study we found that the adolescents referred for weight loss were exceptionally sedentary with activity diaries showing a mean of 10.3 hours/day of seated activity including 4.4 hours of screen time [19]. The physical activity advice provided in the feasibility study was based on the age appropriate Australian Physical Activity Recommendations, which suggests a minimum of 60 minutes per day of moderate and vigorous physical activities [20,21]. Our experience was that young people participating in the feasibility study were reluctant to increase their activity levels based on activity diaries completed over the study period [19]. Therefore, we aim to provide advice that concentrates on a reduction in sedentary behaviour across all groups. Sedentary behaviours will be measured by a physical activity diary and a questionnaire pre- and post intervention to monitor compliance with this strategy.

There is increasing recognition of the need for psychological adjuncts to behavioural approaches in any intervention for childhood weight management [22]. Obesity during childhood and adolescence is associated with negative social and psychological sequelae during a developmentally sensitive period. Overweight children have been found to be at higher risk of low self esteem and low self worth, along with lower levels of perceived competence and social acceptance, compared with healthy weight children [23]. Obese young people are also more likely to be socially isolated and peripheral to social networks than their normal weight peers [24]. Adolescence represents a high risk period for obese youth to experience peer victimisation, such as bullying and teasing due to their weight [25]. In our feasibility study (n = 30) 67% of parents reported that one of the reasons for seeking weight management advice was that their child endured significant teasing/bullying at school, had few friends or difficulty in making friends. Two were refusing to attend regular school reportedly due to weight related victimisation [17]. Evidence also exists that overweight children suffer the psychological distress of early discrimination which may influence their socioeconomic attainment later in life [26].

The Eat Smart protocol will include a unique preparatory phase where we address motivational and perceived self competency issues in a proactive manner by the use of a validated psychological skills programme (FRIENDS *for life*) [27]. This established programme has been demonstrated to reduce anxiety, improve self esteem and develop skills for positive thinking, setting goals and coping with change. The aim of this preparatory phase will be to build self-efficacy and competency together with enhancing emotional resilience and skills training within the family context. Self-esteem, motivation and competency have been reported as predictors of successful weight management in adults [28-30]. Core skills in behavioural modification - goal-setting, problem-solving and self-monitoring will be introduced and these will be developed throughout the dietary intervention. The FRIENDS *for life *program will be conducted in a group setting and will consist of six × 2 hour sessions delivered after school or at weekends, using accredited FRIENDS facilitators. An outline of content is provided in Table 1. Although focused on the adolescent, parents and caregivers join the sessions at the beginning and at the end of each group session so they can help the young person meet their objectives and act in a role of co-therapist. Friends for Life is an established and validated program, staff facilitating the program are either Clinical Psychologists or accredited FRIENDS facilitators [27].

**Table 1:** Outline of FRIENDS for life program

Session	Content Covered
**Week 1**	Introduction to FRIENDS; getting to know you games; group guidelines/expectations; personal goals
**Week 2**	Warm up activity; personal goals; what are my strengths?; self esteem; friendships; verbal and non-verbal communication
**Week 3**	Warm up activity; feelings; relaxation techniques (abdominal breathing, relaxing imagery, muscle relaxation); self-awareness and mindfulness exercises (body clues)
**Week 4**	Warm up activity; red and green (unhelpful and helpful) thinking; problem solving and step planning; support teams; role models
**Week 5**	Warm up activity; communication and conflict styles (assertive, passive or aggressive); managing bullying
**Week 6**	Warm up game; review of FRIENDS; test your knowledge games/quizzes; break up party and certificate presentation from facilitators

### Summary

Intriguing data are available that suggest reducing dietary carbohydrate can lead to weight and body fat loss without the detrimental effects on serum lipid profile. There is a paucity of clinical studies which assess the effectiveness of specific treatment pathways for obese adolescents; no data is available on the relative effect of dietary macronutrient composition on body weight, or how this change affects body composition or metabolic parameters in adolescents. This study will provide RCT evidence on the efficacy of reduced carbohydrate diets compared to low fat diets for treating adolescent obesity. It is the next step in understanding the effect of alteration in dietary macronutrients and their role in body composition and metabolic profile. The novel preparatory phase aims to provide some life skills to ensure that all adolescents entering the study are psychologically prepared for change and free from a range of psychological co-morbidities that can cluster with childhood obesity.

## Methods/Design

Eat Smart is an RCT, which will be reported to be compliant with the CONSORT guidelines [31]. This study design has been approved by the Royal Children's Hospital & Health Service District Ethics Committee (05/02/2008); The University of Queensland Medical Research Review Committee (14/02/2008); West Moreton South Burnett Health Service District Health Research Ethics Committee (25/08/2008) and The Mater Health Services Human Research Ethics Committee (02/02/2009).

**Figure 1:**
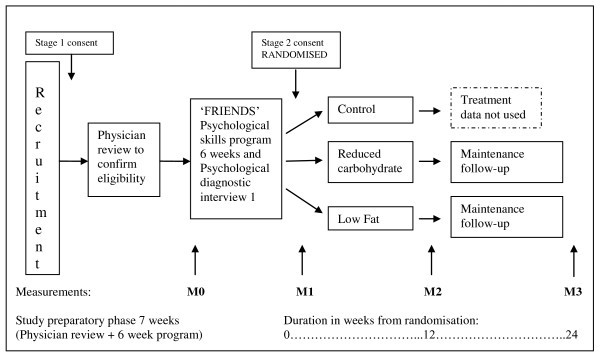
**Diagram of Study Flow**.

Figure 1 shows the study flow. Tables 1 and 2 show an outline of the content of the preparatory phase; 'the 'FRIENDS' program (Table 1) and the intervention phase (Table 2).

**Table 2:** Content Covered in Sessions delivered one to one with therapist

Sessions	Content
Dietary Appointment: Week 0	Rationale of study; individualise food guide; discuss breakfast, lunch and snacks; set SMART goals
Phone Call: Week 1	Check any questions from previous appointment; remind of goals and next appointment
Dietary Appointment: Week 2	Review session 1 goals; trouble shoot problems; discuss food guide for dinner; healthy take-a-ways and eating out; set new SMART goals
Phone Call: Week 4	Check any questions from previous appointment; remind of goals and next appointment
Dietary Appointment: Week 6	Review session 2 goals; trouble shoot problems; label reading workshop; set new SMART goals
Phone Call: Week 8	Check any questions from previous appointment; remind of goals and next appointment
Dietary Appointment: Week 10	Review session 3 goals; trouble shoot problems; sugar in drinks workshop; set new SMART goals
Dietary Appointment: Week 12	Review session 4 goals; trouble shoot problems; Energy balance & maintenance of healthy habits topic; set SMART goals
Phone Call: Week 14	Check any questions from previous appointment; remind of goals and next appointment
*Dietary Appointment: Week 16	Review session 5 goals; trouble shoot problems; Special Occasions; set SMART goals
Phone Call: Week 18	Check any questions from previous appointment; remind of goals and next appointment
*Dietary Appointment: Week 20	Review session 6 goals; trouble shoot problems; Smart Choices topic; Review of previous content; set SMART goals
Phone Call: Week 22	Check any questions from previous appointment; remind of goals and next appointment
Dietary Appointment: Week 24	Review session 7 goals; trouble shoot problems; review energy balance and maintenance topic; set long term SMART goals

SMART = specific, measureable, achievable, realistic, timely

* These may occur as phone reviews for families who cannot attend the hospital.

Overall Eat Smart provides a treatment protocol for use at a tertiary centre to assist obese adolescents seeking weight loss advice to reduce their weight. The first phase will evaluate the relative efficacy of two supported and structured 12 week weight reduction diets and an untreated control group:

(i) reduced carbohydrate (35% carbohydrate, 30% protein, 35% fat)

(ii) reduced fat (55% carbohydrate, 20% protein, 25% fat)

(iii) control - offered the dietary group of their choice (i or ii) after a 12 week waiting period

The primary outcome variable is reduction of BMI z-score. Secondary outcome variables are reduction in body fat and alteration in metabolic profile after 12 weeks. The psychological diagnostic interviews will document the extent and nature of psychological health in obese adolescents. In the second phase, with follow-up to 24 weeks, the relative efficacy of the low fat versus the reduced carbohydrate diet on BMI z-score.

### Hypotheses to be tested

#### After 12 weeks

• There will be a difference in change in BMI z-score between the intervention diets and the control group.

• There will be a difference in the change of mean BMI z-score between the low fat and the reduced carbohydrate group.

• There will be a difference in the change of % body fat, markers of inflammation, insulin resistance and in lipid profile between the reduced carbohydrate group and the low fat group.

#### After 24 weeks

• There will be a difference in mean change in BMI z-score in the low fat group compared to the reduced carbohydrate group.

### Recruitment

All volunteers will be screened via telephone to ensure they meet the inclusion criteria. Participants meeting these criteria will be examined by the study physician (JAB) who will confirm their eligibility, complete a standardised physical examination which includes pubertal staging and measurement of blood pressure.

### Selection Criteria

#### Inclusion

Participants are sought who are aged between 10-17 years; have BMI >90^th ^percentile, as defined by CDC 200 growth charts; can read and understand written instructions in English; and have parents able to give informed written consent.

#### Stage 1 Exclusion

Young people will be excluded on the basis of taking stimulants or psychotropic medication or drugs known to alter body composition or metabolism including insulin sensitisers, glucocorticoids and thyroxin, those with obesity associated with a medical condition such as Down's Syndrome and those following severely restricted diets, such as those for complex food allergies.

#### Stage 2 Exclusion

Participants will undertake the Anxiety Disorder Interview Schedule (ADIS) conducted by a Clinical Psychologist after completion of the psychological skills programme [27] prior to randomisation (Measurement1) and at Measurement 2 to investigate any alteration in psychological functioning post intervention (see figure 1).

In the event that the psychological ADIS interview suggests the presence of significant and clinically relevant levels of anxiety, depression, dysthymia, conduct disorders, eating disorders, psychotic disorders, attention deficit disorders and obsessive compulsive disorders, young people will be excluded and referred for clinical psychological services in conjunction with their referring doctor. This will be facilitated via the study and psychological sessions will be arranged through a Mental Health Care Plan, therefore with no out of pocket costs to the family.

This will ensure all volunteers must score below the cut offs for clinical diagnosis on the following validated questionnaires: The Strengths and Difficulties questionnaire[32]; the Depression, Anxiety and Stress Scale [33]; the Spence Children's Anxiety scale [34]; and the Children's Depression Inventory [35].

### Ethical considerations

Lack of treatment for the obese child should not be an option as weight gain in those not treated is unrelenting. A recent meta-analysis of randomised controlled trials for treatment of overweight in children clearly demonstrates that control groups whether wait listed or only given written information consistently gained weight (2.1% increase in % overweight post treatment and 2.7% post follow-up) [36]. This increase in weight in control groups raises ethical issues around having a control group, especially in a clinical setting with children presenting for assistance with weight loss. We received ethical permission to delay treatment in a control group for 12 weeks after which we offer the program of their choice. These ethical issues will limit the ability to design trials with a control group remaining without intervention for lengthy study duration, especially in treatment seeking adolescents.

### Interventions

A 12-week dietary intervention will be delivered by paediatric dietitians with training in behaviour modification. To ensure intervention fidelity across the two treatment arms, a standardised manual including protocols, procedures and participant materials will be used. All intervention families will be offered face to face tailored advice in the active weight loss phase in weeks 0, 2, 6, 10 and 12. Further dietary counselling will be provided by telephone in weeks 1, 4, 8 and 11. These follow a pre-determined outline to ensure consistency across treatment arms. The dietetic intervention is deliberately intensive in keeping with recent recommendations for best practice dietary management of obesity [37]. It is recognised that dietary advice must be delivered using a client-centred approach and motivational interviewing in tandem with behavioural components including problem-solving, goal-setting and self-monitoring [37,38]. We will use two strategies to achieve these goals. First, the use of experienced paediatric dietitians to deliver the dietary advice and secondly, by preparing the adolescents and equipping them with the skills they need via the FRIENDS programme prior to the RCT commencing. The diets are to be used within the family context. We are cognisant of the fact that many parents are overweight (>90% in our feasibility study) and thus will adopt a 'treat the family' approach. Written information (diet sheets, meal plans, recipe ideas and shopping lists) which supports each of the diets have been developed. An outline of each session is summarised in Table 2.

### Energy prescription

A tailored individual energy prescription for the intervention diets will be based on a 20% energy reduction compared to estimated energy requirements, which will be measured resting energy expenditure and physical activity level (PAL) derived from a 4 day activity diary. The 12 week intervention is designed to be an active weight loss phase. During the 'Maintenance Phase' (weeks 13-24), the goal is weight stability and maintenance of healthy behaviours.

### Intervention diets

Both dietary plans use a structured approach with a plate portion system to assist with portion sizes for the evening meal. The dietary approaches utilised in the intervention have been tested within the feasibility stage of this project. The plate templates (TEMPlate™) have necessarily different size sections for the low fat and the reduced carbohydrate diets to enable the desired macronutrient composition of the meal to be achieved. In addition, a lunch box is provided with lists of foods for snacks and lunch that can be swapped by the individual. This enables food items for the day to be packed and given to the child or kept in the fridge if the parent is not around at meal or snack times. We have found that the benefits of using this structured system are that both the parent and child know what foods are allocated through the day for most meals, the plate template assists with portion control at the evening meal and the 'food swapping' system allows flexibility with food choices over a long period of time, whilst controlling the macronutrient distribution of the diet. The plate template is made of durable plastic and is dishwasher proof.

### Reduced carbohydrate

A reduced carbohydrate (35% of energy) diet with moderate-high protein intake (30% of energy) and fat (35% of energy) with unsaturated sources of fat strongly encouraged. This level of carbohydrate restriction is not sufficient to induce ketosis [39]. Complex carbohydrates are spread over the day and given in a structured amount at each meal or snack with an emphasis on those with a low glycaemic index. Protein is increased with the intent to give greater satiety.

### Low fat

A low fat diet containing (25% of energy from fat) with a higher carbohydrate intake (55% of energy) with complex carbohydrate sources encouraged and a moderate protein intake (20% of energy). This dietary approach recommends low fat foods and snacks and increases fruit and vegetable intake.

### Control group

The control group will be offered treatment for 24 weeks after the formal control period (of 12 weeks).

### Physical activity and sedentary behaviours

The intervention groups will receive the Australian National Health and Medical Research Councils 'Get out and get active' booklet. Emphasis will be placed on a reduction in sedentary behaviours. Activity will be measured by qualitative comparison of the measures from The Adolescent Physical Activity Recall Questionnaire [40] and a 4 day physical activity diary which will be completed at M1 and M2 (all groups).

### Assessments and Measurement instruments

The laboratory technician conducting body composition measurements will be blinded to group allocation. The following assessments will be completed at M1 and M2 unless otherwise stated (see figure 1).

### Anthropometry and body composition

These measurements will be undertaken by the body composition laboratory technician using standard operating procedures. Height, weight, waist and hip circumferences will be measured. BMI z-score will be calculated by the LMS method [41] using CDC reference data. Body composition will be assessed by 3 methods: air displacement plethysmography (BodPod™), total body potassium (TBK) and bioelectrical impedance (Bodystat 1500MDD). These measures are non-invasive and we have validated these for use in obese adolescents [42].

### Liver function and plasma lipid profiles

Fasting blood samples (10 ml) will be taken by trained phlebotomists at M1 and M2. Liver function tests (including total protein, albumin, total bilirubin, liver function enzymes (ALP, GGT, ALT and AST) and lipid profiles (including cholesterol fractions and triglycerides) will be measured using an automated Clinical Chemistry Analyser.

### Insulin resistance

Fasting glucose and insulin will be measured using an automated Clinical Chemistry Analyser. The homeostatic method will be used to calculate insulin resistance and beta cell function.

### Adipokines and inflammatory markers

Adipose tissue is not only a site of energy storage, but also an active endocrine organ secreting a number of hormones and cytokines that play critical roles in body energy homeostasis and metabolism [43]. We will measure the following adipokines and cytokines at M1 and M2: leptin, resistin, adiponectin, interleukin-6, TNFα, CRP, plasminogen activator inhibitor 1 (PAI1) and soluble ICAM-1 in plasma using a multiplexed immunoassay, analysed on the Luminex 100IS.

### Diet and activity

Participants will be asked to complete a 4 day food and activity records at M1 and M2. Food records will be analysed using a standard nutrient analysis package (FoodWorks). Physical activity habits will be assessed using the diary and the Adolescent Physical Activity Recall Questionnaire [40].

### Allocation to groups

Treatment allocation will depend on gender and Tanner Stage (pubertal stage). Weighted randomisation will be used so that the participants will be allocated to the treatment group that minimises the gender/Tanner Stage imbalance between groups with a probability of 0.8 and will be conducted by the study statistician (RSW). Participants will be informed as to which group they have been assigned at their baseline assessment and if they are in the active diet groups, an immediate appointment with a study dietitian will be made. The control group will be given an appointment to attend in 12 weeks time when they will be offered the intervention treatment of their choice.

### Compliance and retention strategies

Compliance to the dietary program will be monitored by the use of 4 day food dairies throughout the intervention and at each measurement point. To try and reduce attrition, volunteer families will have parking cost at the hospital paid and during the initial 12 week period there is frequent and intense therapist contact. In the feasibility study we documented a mean increase in plasma urea in the reduced carbohydrate group (not statistically significant) but it is suggestive that dietary protein shifts in the direction indicated by the food diaries. We will measure serum changes in urea and urates across groups over time and also measure serum vitamin B12 as a surrogate marker of protein intake as this has been shown to increase in adults on high protein diets [15]. We recognise this method has not been validated in children.

### Sample size

Our sample size calculations were based on our primary comparison, the difference in change in BMI z-score between the low fat and reduced carbohydrate groups. In our feasibility study (n = 30), the mean (SD) of BMI z-score changes over 12 weeks were -0.20 (0.17) and -0.11 (0.08) for the low fat and reduced carbohydrate groups respectively. In order to observe a clinically important difference in change of BMI z-score between the two intervention groups of 0.09, with α = 0.05 and power of 80%, and assuming standard deviation of 0.12, we require 29 individuals in each intervention group to complete the study. Assuming that 20% of participants will drop out of the study (in our feasibility study our attrition rate was 17%), we will enrol 35 individuals in each of the diet intervention groups. If 12 participants enrolled to the control group complete the 12 week wait-list period, we will be able to detect a difference of change in BMI z-score of 0.13 between the control group and either of the diet intervention groups (α = 0.05, power = 80%). If we assume 20% of control-group participants will drop out of the study, we will need to enrol 15 individuals in the control group.

### Statistical Analysis Plan

Primary analysis will use the intention to treat principle, assuming return to baseline values for non-completers. Differences between participants who completed and withdrew will be assessed using t tests for continuous variables and Fisher's Exact Test for categorical variables. We shall examine weight loss over time using a mixed-effects model with a random intercept and slope for each participant. This method controls for the non-independence in results from the same participant and allows for heterogeneity between participants. Potential co-variates (gender, Tanner Stage, baseline weight) will be assessed, and, if appropriate, incorporated into the model. We will examine the effects of losses of body weight and body fat on clinically important markers of disease risk (lipids, blood pressure, waist: hip ratio, insulin resistance) using analysis of covariance. Data generated from the psychometric questionnaires (self-concept, anxiety, self-esteem and quality of life) will be used as secondary outcome variables to examine the extent and nature of psychological distress in obese adolescents pre-treatment and mixed-effects models will be used to examine changes in these parameters and their relationship with weight and waist: hip ratio change. Statistical significance will be based on two-tailed tests, with p < 0.05 considered significant.

## Discussion

There is a lack of clinical studies which assess the effectiveness of specific treatment pathways for obese adolescents. In particular there is no information as to the effect of how alteration of the carbohydrate content of the diet will change body composition or metabolic parameters. This study protocol will contribute also to our understanding of body composition of obese adolescents and the effect of weight loss on different body compartment changes.

Our feasibility study suggested that tailored energy prescription and structured advice was the preferred option for treatment seeking adolescents and their families. In particular the need for information on how much to eat i.e. portion size was requested. If the reduction in dietary carbohydrate has no adverse effects on cardiovascular risk factors, the option to decrease carbohydrate would broaden the choice of dietary patterns based on preference of the adolescent and their family that could be offered in practice.

This study has a unique preparatory psychological preparatory phase and screening for psychological disturbance prior to randomisation which ensures that all subjects enter the study without any clinical levels of psychological diagnosis. The additional measurement of eating behaviours and other psychological parameters is designed to monitor any risk to psychological health associated with the intervention. The relatively short time frame allows biochemical parameters and psychological parameters to be measured before taking the study into a longer duration.

This study is designed to address the gap in the evidence base for optimal weight management interventions in obese adolescents in a clinical setting and to examine effect of macronutrient manipulation on body composition and cardiovascular risk markers in adolescents presenting for weight management. It is designed as a treatment protocol integrating psychological support with dietary change. It incorporates group based activity, individual face to face counselling and telephone support in assisting adolescents and their families with weight management.

## Competing interests

The authors declare that they have no competing interests.

## Authors' contributions

HT and JAB took the lead in designing the study, KAB, LAD, PSWD and JC contributed to the study design and grant preparation, PB contributed the modification of the 'FRIENDS for life' program; RSW is responsible for the statistical analysis plan. All authors have approved the final version of this paper for publication.

## Pre-publication history

The pre-publication history for this paper can be accessed here:

http://www.biomedcentral.com/1471-2458/10/464/prepub
